# Pathway Instability Is an Effective New Mutation-Based Type of Cancer Biomarkers

**DOI:** 10.3389/fonc.2018.00658

**Published:** 2019-01-04

**Authors:** Marianna A. Zolotovskaia, Maxim I. Sorokin, Sergey A. Roumiantsev, Nikolay M. Borisov, Anton A. Buzdin

**Affiliations:** ^1^Department of Oncology, Hematology and Radiotherapy of Pediatric Faculty, Pirogov Russian National Research Medical University, Moscow, Russia; ^2^Oncobox Ltd., Moscow, Russia; ^3^The Laboratory of Clinical Bioinformatics, I.M. Sechenov First Moscow State Medical University, Moscow, Russia; ^4^Omicsway Corp., Walnut, CA, United States; ^5^The Laboratory of Systems Biology, Shemyakin-Ovchinnikov Institute of Bioorganic Chemistry, Moscow, Russia

**Keywords:** cancer, DNA mutation, molecular pathways, biomarker, pathway instability

## Abstract

DNA mutations play a crucial role in cancer development and progression. Mutation profiles vary dramatically in different cancer types and between individual tumors. Mutations of several individual genes are known as reliable cancer biomarkers, although the number of such genes is tiny and does not enable differential diagnostics for most of the cancers. We report here a technique enabling dramatically increased efficiency of cancer biomarkers development using DNA mutations data. It includes a quantitative metric termed Pathway instability (PI) based on mutations enrichment of intracellular molecular pathways. This method was tested on 5,956 tumor mutation profiles of 15 cancer types from The Cancer Genome Atlas (TCGA) project. Totally, we screened 2,316,670 mutations in 19,872 genes and 1,748 molecular pathways. Our results demonstrated considerable advantage of pathway-based mutation biomarkers over individual gene mutation profiles, as reflected by more than two orders of magnitude greater numbers by high-quality [ROC area-under-curve (AUC)>0.75] biomarkers. For example, the number of such high-quality mutational biomarkers distinguishing between different cancer types was only six for the individual gene mutations, and already 660 for the pathway-based biomarkers. These results evidence that PI value can be used as a new generation of complex cancer biomarkers significantly outperforming the existing gene mutation biomarkers.

## Introduction

Cancer is a multifactorial disease which is conditioned by alterations arising from biological, chemical, radiological impacts, as well as inherited. Tumor transformation is characterized by frequent accumulation of genetic mutations (1). The pivotal initiating role here belongs to DNA damage and genome instability (2, 3). The resulting combinations of gene mutations driving cancer development vary dramatically among different cancers types and individual patients (4).

Recently, high throughput studies of cancer genomes were initiated to identify mutation enrichment specific for the different cancer types. For example, the large scale projects like Wellcome Trust Sanger Institute's Cancer Genome Project, the International Cancer Genome Consortium (ICGC), The Cancer Genome Atlas (TCGA) showed very high molecular heterogeneity of cancer, not only between different cancer types, but also among the individual tumors of the same type (5–8). This allowed to considerably advance current understanding of carcinogenetic molecular mechanisms by documenting complete or near-complete landscapes of pathological somatic mutations including base substitutions and gene fusions. Many of the alterations revealed appeared promising for molecular cancer diagnostics in order to improve and personalize the treatment regimens (9, 10).

Identification of informative and robust genetic markers of cancer is one of the major tasks of the contemporary biomedicine. Many reports have been published featuring cancer-specific mutations and gene fusions, as well as epigenetic alterations (11–13). Some of them are already widely used in clinical practice as the biomarkers, but the problem of finding new relevant and informative cancer markers with higher sensitivity and specificity is largely unsolved (10, 14). Further accumulation of cancer type- and condition-specific biomarkers can be a key to a more effective, personalized treatment (15).

Despite recent success in high throughput analysis of molecular basis for cancer transformation, traditionally the focus is being made on the roles of the individual genes (16, 17). However, this approach cannot always explain tumor development in a comprehensive way. Apparently, this is most probably due to the mode of gene functioning as the nodes of molecular pathways, where roles of individual genes are highly interconnected and frequently interchangeable (18).

Previously, the analysis of molecular pathways at the level of gene expression was successfully applied for cancer investigations (19–21). Several approaches for measuring molecular pathway activities were proposed for the expression data at both mRNA, protein and microRNA levels (22–27). The extent of pathway activation, so called *pathway activation strength (PAS)*, is a cumulative value aggregating relative expression levels of the enclosed gene products in relation with their functional roles in a pathway (28).

Interestingly, for most of the cancer types investigated the molecular pathways were shown to be stronger expression biomarkers of cancer than the individual genes (29). *PAS* was also proven to be more stable and experimental platform-independent metric than the individual gene expression patterns (30). This property appeared to be fundamental and linked with the ability to aggregate individual gene expression levels, thus decreasing experimental errors, as modeled in a recent investigation (25). *PAS* biomarkers are also used for predicting efficiencies of target cancer drugs in the ongoing clinical trials (24). However, to our knowledge, this type of quantitative pathway approach was never applied before for the mutation data, including human cancers.

Here, for the first time, we propose a new type of molecular biomarkers based on DNA mutation impacts on the molecular pathways. We introduced a quantitative metric termed *Pathway instability* (*PI*) proportionate to the relative number of mutated genes in a pathway and developed a specific bioinformatic algorithm for quantization thereof. Using high throughput gene mutation profiles, we identified affected molecular pathways that specifically define the major human cancer types. We took cancer somatic mutation data published in the TCGA project for 5,956 patients representing 15 different cancer types. Totally, we screened 2,316,670 mutations in 19,872 genes and 1,748 molecular pathways. The robustness of mutation-based molecular pathway approach dramatically exceeded that for the individual gene biomarkers. This trend was also reproducible when only truncating mutations were considered for *PI* calculations, thus confirming consistency of the new method. Finally, we provide a list of 660 novel robust cancer type-specific pathway mutation biomarkers.

## Materials and Methods

### DNA Mutation Data

The source DNA mutation dataset was obtained from the database of COSMIC (the Catalog Of Somatic Mutations In Cancer) project (31). We downloaded the verified somatic mutations data from COSMIC website, database version 76 (32). The initial dataset contained 6,651,236 mutation records for 20,528 genes from 19,434 individual tumor samples of 37 primary localizations. For statistical consistence, we took only those tumor localizations having at least 100 tumor samples profiled during *The Cancer Genome Atlas* (TCGA) project (33). The TCGA mutation profiles were selected because they represented the largest collection of uniformly treated biosamples profiled using the same deep sequencing platforms (34). Totally, we analyzed 2,316,670 mutations in 5,956 tumor genetic profiles corresponding to 15 primary localizations: breast, central nervous system, cervical, endometrium, ovaries, prostate, kidney, urinary tract, liver, hematopoietic and lymphoid tissue, stomach, large intestine, lung, thyroid, and skin (Supplementary Table 1). The database accession numbers of 5,956 samples are given in Supplementary Table 3.

In parallel, for the additional analysis we selected a fraction of gene truncating mutations that possibly lead to the loss of gene function. We meant truncating mutations as those having the following labels in COSMIC description: “Deletion-Frameshift,” “Insertion-Frameshift,” “Complex-frameshift,” “Substitution-Nonsense.” Totally, we selected 161,760 truncating mutations in 5,297 tumor samples corresponding to the same 15 cancer types (Supplementary Table 2). The database accession numbers of 5,297 samples are given in Supplementary Table 4.

### Molecular Pathways

The structures of 3,121 molecular pathways were taken from the following public databases: Reactome (35), NCI Pathway Interaction Database (36), Kyoto Encyclopedia of Genes and Genomes (37), HumanCyc (38), Biocarta (39), Qiagen (40). For all the pathways, the gene contents were extracted and cataloged. For further analyses, we pre-selected 1,748 molecular pathways each including at least 10 gene products.

### Principal Component Analysis

The Principal component analysis (PCA) was performed with package made 4 in R.

### Clustering Dendrograms

For clustering, we used Ward's criterion and Ward.d2 algorithm (41) for the gene- (*nMR*) and pathway- (*PI*) specific mutation metrics.

## Results

In this study, we applied quantitative molecular pathway approach to human cancer DNA mutation data. We developed algorithm for quantization of mutational impact on molecular pathways and applied it for screening of 1,748 human pathways. The DNA mutation data was extracted from the Catalog Of Somatic Mutations In Cancer (COSMIC) database (32). For the reasons of statistical significance, we analyzed here only the tumor localizations having at least 100 complete exome sequencing-profiled tumor samples, totally fifteen primary localizations and 5,956 individual tumor specimens (Supplementary Table 1). For parallel analysis, we also selected a subset of 161,760 truncating mutations for 5,297 tumor samples of the same cancer types (Supplementary Table 2).

### Pathway Instability (PI) Scoring

*Pathway instability* (*PI*) scoring for a molecular pathway depends on the mutation frequencies in the genes participating in this pathway. To assess mutation burden of the *individual genes*, we introduced *Mutation rate* (*MR*) value calculated according to the formula:

$$\text{M}{\text{R}}_{\text{n}}=\frac{\text{N}\;\text{mut}(\text{n},\text{g})}{\text{N}\;\text{samples}\;(\text{g})}$$

where *MR*_*n*_ is the *Mutation rate* of a gene *n*; *N mut(n,g)* is the total number of mutations identified for a gene *n* in a group of samples *g*; *N samples (g)* is the number of samples in a group *g*. However, the *MR* values strongly positively correlated with the lengths of gene coding DNA sequences (CDS)s, Spearman correlation was 0.798 for all mutations and 0.629 for truncating mutations, *p* < 2.2e-16 in both cases (Supplementary Images 1A,C), most probably because larger genes had higher probabilities to accumulate mutations.

To avoid the bias linked with the CDS lengths, we next introduced a *Normalized mutation rate* (*nMR*) value expressed by the formula:

$$\text{nM}{\text{R}}_{\text{n}}=\frac{1000^{*}\text{M}{\text{R}}_{\text{n}}}{\text{Length}\;\text{CDS}\;(\text{n})\;},$$

where *nMR*_*n*_ is the *Normalized mutation rate* of a gene *n*; *MR*_*n*_ is the *Mutation rate* of a gene *n*; *Length CDS(n)* is the length of CDS of a gene *n* in nucleotides.

In contrast to the previous metric, *nMR* did not correlate with the size of CDS for the respective genes (Supplementary Images 1B,D; rank correlation 0.151 for all mutations and 0.024 for truncating mutations). nMR scores calculated using all mutations for each tumor sample are listed in Supplementary Data Sheet 1, nMR scores calculated based on truncating mutations only are listed in Supplementary Data Sheet 2.

We next calculated *Pathway instability (PI)* scores for every pathway to estimate their relative enrichments by cancer-specific mutations. *Pathway instability* is expressed by the formula:

$$\begin{array}{l} {\text{P}\text{I}}_{\text{p}}=\frac{\sum_{\text{n}}\text{n}{\text{M}\text{R}}_{\text{n}}{\text{P}\text{G}}_{\text{p},\text{n}}}{{\text{N}}_{\text{p}}} \end{array}$$

where *PI*_*p*_ is *Pathway instability* score for a pathway *p*; *nMR*_*n*_ is the *Normalized mutation rate* of gene *n*; *PG*_*p*.*n*_ is pathway-gene indicator that equals to one if gene *n* belongs to pathway *p*, or equals to zero if gene *n* doesn't belong to pathway *p*; *N*_*p*_–total number of gene products that belong to pathway *p*. Unlike previous metrics for pathway activation scoring based on gene expression data, the current equation for *PI* calculation doesn't have coefficients defining activator or repressor molecular roles of genes participating in a molecular pathway under investigation (28). This has been done because for most of mutations their functional roles (neutral, repressing or activating gene function) remain unclear. *Pathway instability* (*PI)* (calculation also doesn't utilize logarithmation of *nMR* scores during summation because the number of mutations in tumor is not lower than in the reference normal tissue.

### Cancer Type-Specific Pathway Instability (PI) Mutation Signatures

Totally, we calculated *PI* scores for 1,748 molecular pathways in 5,956 tumor samples representing fifteen primary localizations: breast, central nervous system, cervical, endometrium, ovaries, prostate, kidney, urinary tract, liver, hematopoietic and lymphoid tissue, stomach, large intestine, lung, thyroid, and skin for all mutations (Supplementary Data Sheet 3) and in 5,297 tumors for only truncating mutations (Supplementary Data Sheet 4).

**Figure 1:**
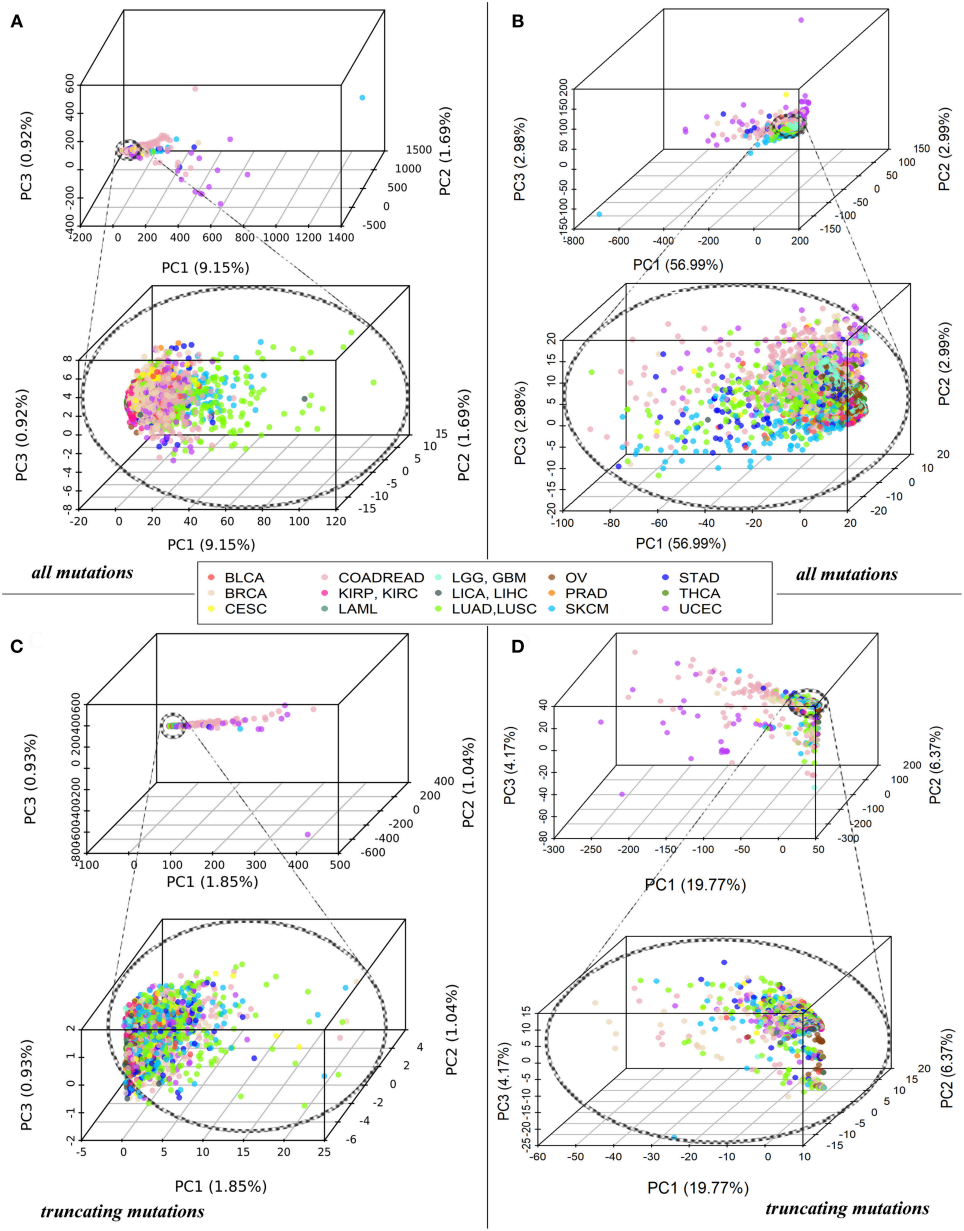
**(A)** PCA of *Normalized mutation rate (nMR)* patterns based on all mutations for 5,956 samples representing 15 primary human tumor localizations, reflected by the color key. Each point on the plot represents one tumor sample. Abbreviations for the cancer types: BRCA, breast invasive carcinoma; LGG, brain lower grade glioma; GBM, glioblastoma multiforme; CESC, cervical squamous cell carcinoma and endocervical adenocarcinoma; UCEC, uterine corpus endometrial carcinoma; LAML, acute myeloid leukemia; KIRP, kidney renal papillary cell carcinoma; KIRC, kidney renal clear cell carcinoma; COADREAD, colorectal cancer; LICA, liver cancer; LIHC, liver hepatocellular carcinoma; LUAD, lung adenocarcinoma; LUSC, lung squamous cell carcinoma; OV, ovarian serous cystadenocarcinoma; PRAD, prostate adenocarcinoma; SKCM, skin cutaneous melanoma; STAD, stomach adenocarcinoma; THCA, thyroid carcinoma; BLCA, bladder urothelial carcinoma. **(B)** PCA of *Pathway instability* (*PI*) patterns based on all mutations for the same samples. **(C)** PCA of *Normalized mutation rate (nMR)* patterns based on the truncating mutations for 5,297 tumor samples. **(D)** PCA of *Pathway instability* (*PI*) patterns based on the truncating mutations for 5,297 tumor samples.

Each tumor sample was characterized by a complete set of *Normalized mutation rate (nMR)* values for all individual genes and by the *Pathway instability* (*PI)* values for 1,748 molecular pathways. As shown by the principal component analysis (PCA), the complete sets of 19,872 *nMR* biomarkers and 1,748 PI biomarkers could not distinguish between the tumor localization types (Figures 1A,B). The PCA revealed no significant differences for the different cancer types both at the gene-based and pathway-based levels, although pathway-based approach covered most of the variation by the first component, unlike gene-based level of data analysis, where dramatically lower proportion of variation was covered. Similar figure was seen for the fraction of truncating mutations, where the variation of first principal component did not exceed 1.85% for genes and 19.77% for pathways (Figures 1C,D). The most likely reason for lack of tumor type-specific clustering may be the redundancy of features at high sparsity of the mutation data.

However, many molecular pathways had characteristic PI scores that were clearly distinctive of the different tumor types, as shown by the high *area under the ROC curve* (AUC) values. The AUC value is the universal biomarker robustness characteristics depending on its sensitivity and specificity(42). It varies from 0.5 till 1 and positively correlates with the quality of a biomarker. The AUC discrimination threshold is typically 0.7 or 0.75. The parameters with greater AUC are considered good-quality biomarkers, and vice-versa (43). This statistical approach is also applicable to mutation research in human cancer (44–46). We performed the ROC AUC test in two ways: (*i*) for comparing every separately taken tumor type (localization) vs. all other tumors, and (*ii*) for all possible pairwise comparisons among the tumor types.

**Figure 2:**
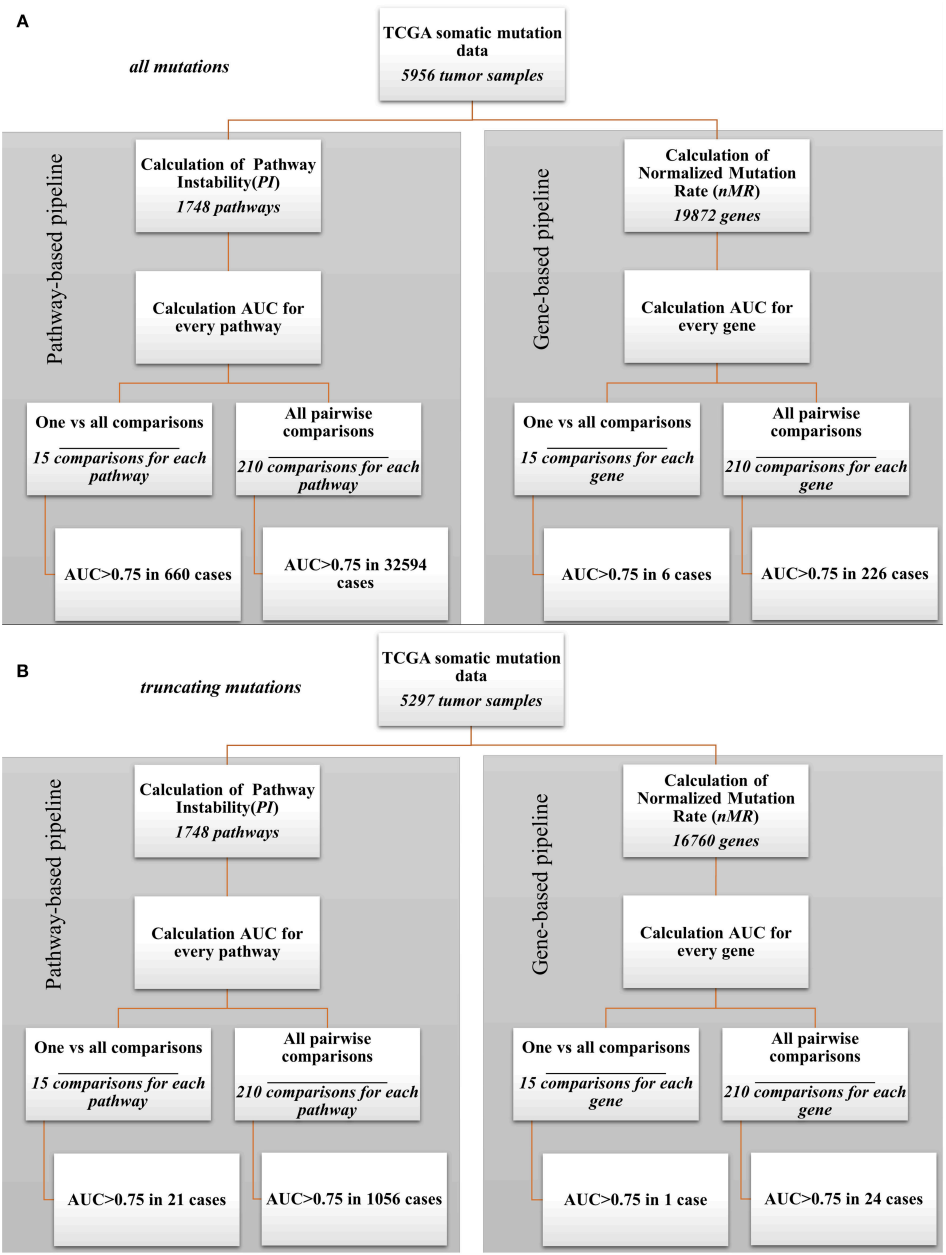
Bioinformatic comparison of quality for pathway- and gene-based mutation biomarkers: **(A)** for all mutations; **(B)** for truncating mutations.

In parallel, the same AUC tests were performed also for every gene *nMR* characteristics of every sample. The tests were performed for all mutations and in parallel—for only truncating mutations (Supplementary Tables 5, 6, respectively). The data analysis pipeline is schematized on Figure 2. In this way, we could compare the biomarker potentials of the individual gene mutations (*nMR*) with the aggregated pathway-based mutation characteristics (*PI*).

Our analysis revealed a dramatic advantage of the pathway based (*PI*) compared to gene based (*nMR*) approach in finding good quality biomarkers in all types of the comparisons made. For example, for the analyzes when one tumor localization was compared against fourteen others, the total number of good quality (AUC>0.75) biomarkers was 660 for all mutations and 21 for truncating mutations for the pathways (*PI*), compared to only six for all mutations and one for truncating mutations for the individual genes (*nMR*). Similarly, for the pairwise comparisons we identified totally 32,594 good quality *PI* biomarkers vs. only 226 *nMR* biomarkers for all mutations (Figure 2A). For the truncating mutations, we discovered in pairwise comparisons 1,056 good quality *PI* biomarkers vs. only 24 *nMR* biomarkers (Figure 2B). Provided that the initial number of potential pathway biomarkers (1748) was one order of magnitude lower than the number of gene biomarkers (19,872 for all mutations and 16,760 for truncating mutations), this further strengthens the advantage of a *PI*-based approach.

### Cancer Type Specific Biomarkers

**Figure 3:**
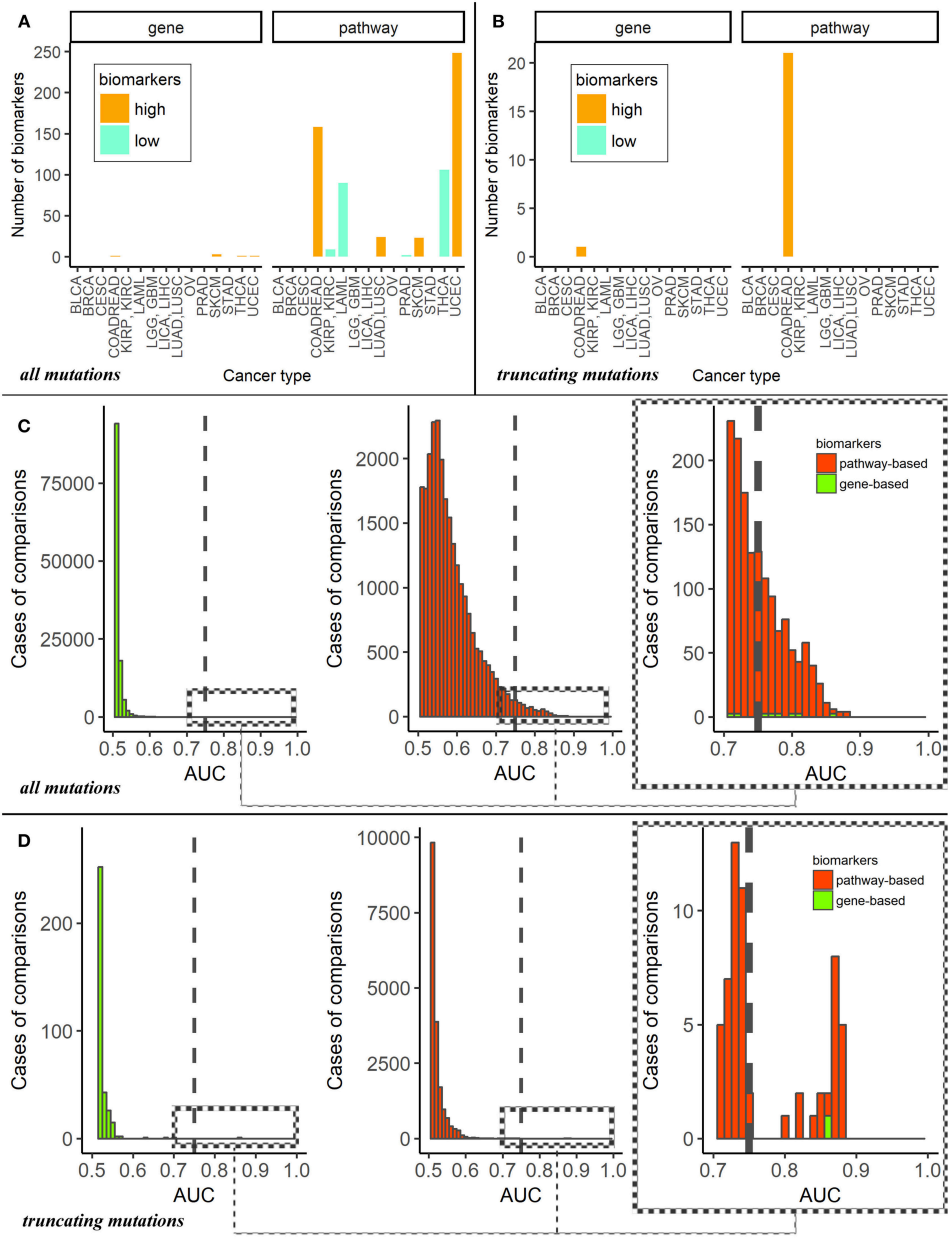
**(A)** Numbers of mutation marker genes and molecular pathways in “one vs. all” cancer type comparisons for all mutations. The cancer types are abbreviated as follows: BRCA, breast invasive carcinoma; LGG, brain lower grade glioma; GBM, glioblastoma multiforme; CESC, cervical squamous cell carcinoma and endocervical adenocarcinoma; UCEC, uterine corpus endometrial carcinoma; LAML, acute myeloid leukemia; KIRP, kidney renal papillary cell carcinoma; KIRC, kidney renal clear cell carcinoma; COADREAD, colorectal cancer; LICA, liver cancer; LIHC, liver hepatocellular carcinoma; LUAD, lung adenocarcinoma; LUSC, lung squamous cell carcinoma; OV, ovarian serous cystadenocarcinoma; PRAD, prostate adenocarcinoma; SKCM, skin cutaneous melanoma; STAD, stomach adenocarcinoma; THCA, thyroid carcinoma; BLCA, bladder urothelial carcinoma. **(B)** Numbers of mutation marker genes and molecular pathways in “one vs. other cancer types” comparisons for truncating mutations only. **(C)** AUC distributions of pathway- and genes-based mutation biomarkers for all mutations. Cut-off AUC level of high-quality biomarkers is set 0.75. AUC were obtained as the result of “one vs. others” comparisons. **(D)** AUC distributions of pathway—and genes-based mutation biomarkers for truncating mutations only. Cut-off level of high-quality biomarkers is set 0.75. AUC were obtained in “one vs. other cancer types” comparisons.

For the dataset of all mutations, the cancer type-specific six gene mutation biomarkers were *APC* for colorectal cancer, *PTEN* for endometrial cancer, *BRAF* for thyroid cancer and *MUC16, DNAH5, TTN* for cutaneous melanoma. These genes were previously linked with the respective cancer types in the literature (47–50), but the overall number of six biomarkers may seem negligible provided they were obtained for fifteen comparisons (Figure 2A). In contrast, the pathway approach returned here as much as 660 reliable biomarkers representing 428 pathways. Different localizations had markedly different numbers of marker pathways (Figure 3A). Gene and pathway mutation biomarkers could be found for four and eight tumor localizations, respectively. In the case of truncating mutations, we found gene and pathway biomarkers for colorectal cancer only (Figures 3B,D).

For the first time, we provide here the list of tumor type-specific pathway biomarkers based on all mutation for eight localizations investigated: colorectal, kidney, non-small cell lung, prostate, thyroid cancers, hematological malignancies, cutaneous melanoma and uterine corpus endometrial carcinoma (Supplementary Table 7). The list of colorectal cancer specific pathway biomarkers obtained using truncating mutations is shown on Supplementary Table 8.

Despite the large number of good-quality pathway biomarkers (Figures 3A,C), all of them were applicable only for eight localizations of the fifteen totally investigated. Colorectal cancer and endometrial carcinoma had maximum number of pathway-based biomarkers. It should be noted that characteristic tumor type-specific PI scores could be either higher or lower than the average values for other cancer types, thus resulting in “high” or “low” biomarkers (Figure 3A).

Using total pool of mutations, we next identified molecular pathways that were frequently mutated in all the cancer types under study. To this end, we selected 1,145 pathways having AUC < 0.7 in all tumor types (Supplementary Table 9) and intersected them with the list of top 10% pathways sorted according to the average *PI* values. The selected short list contained 18 pathways that were most frequently mutated in all cancer types (Table 1).

**Table 1:** Intersection of top 10% molecular pathways by average PI and molecular pathways with AUC < 0.7 for all cancer types.

**#**	**Pathway name (according to the source pathway database)**	**PI**	**Reference**
1	NCI Aurora A signaling Pathway (protein catabolic process)	0.19	http://apps.pathwaycommons.org/pathways?uri=http%3A%2F%2Fpathwaycommons.org%2Fpc2%2FPathway_83968ff327912d4d5a0ee5f31d27adf9
2	biocarta double stranded RNA induced gene expression Main Pathway	0.19	http://amp.pharm.mssm.edu/Harmonizome/gene_set/double+stranded+rna+induced+gene+expression/Biocarta+Pathways
3	biocarta role of *BRCA1 BRCA2* and *ATR* in cancer susceptibility Pathway (DNA replication termination)	0.18	http://amp.pharm.mssm.edu/Harmonizome/gene_set/role+of+brca1+brca2+and+atr+in+cancer+susceptibility/Biocarta+Pathways
4	biocarta *p53* signaling Main Pathway	0.18	http://amp.pharm.mssm.edu/Harmonizome/gene_set/p53+signaling+pathway/Biocarta+Pathways
5	*NGF* Pathway Apoptosis	0.17	https://www.qiagen.com/us/shop/genes-and-pathways/pathway-details/?pwid=320
6	*BRCA1* Pathway Mismatch Repair	0.16	https://www.qiagen.com/us/shop/genes-and-pathways/pathway-details/?pwid=68
7	*ATM* Pathway G2-Mitosis progression	0.16	https://www.qiagen.com/us/shop/genes-and-pathways/pathway-details/?pwid=46
8	*ATM* Pathway G2 M Checkpoint Arrest	0.16	https://www.qiagen.com/us/shop/genes-and-pathways/pathway-details/?pwid=46
9	biocarta tumor suppressor *ARF* inhibits ribosomal biogenesis Main Pathway	0.16	http://software.broadinstitute.org/gsea/msigdb/cards/BIOCARTA_ARF_PATHWAY
10	biocarta *ATM* signaling Main Pathway	0.14	http://software.broadinstitute.org/gsea/msigdb/geneset_page.jsp?geneSetName=BIOCARTA_ATM_PATHWAY
11	NCI Hypoxic and oxygen homeostasis regulation of *HIF1* alpha Main Pathway	0.12	http://www.pathwaycommons.org/pc/record2.do?id=517145
12	*HIF1* Alpha Pathway *NOS* Pathway	0.11	https://www.qiagen.com/us/shop/genes-and-pathways/pathway-details/?pwid=223
13	*HIF1*Alpha Pathway *VEGF* Pathway	0.09	https://www.qiagen.com/us/shop/genes-and-pathways/pathway-details/?pwid=223
14	*HIF1*Alpha Pathway Gene Expression via *JUN CREB3*	0.09	https://www.qiagen.com/us/shop/genes-and-pathways/pathway-details/?pwid=223
15	Lipoxins Influence on Cell Growth and Proliferation	0.05	http://pathwaymaps.com/maps/2690
16	NCI Validated transcriptional targets of TAp63 isoforms Pathway (Pathway degradation of TP63)	0.03	http://www.pathwaycommons.org/pc/record2.do?id=517011
17	NCI Validated transcriptional targets of TAp63 isoforms Pathway (Metastasis)	0.02	http://www.pathwaycommons.org/pc/record2.do?id=517011
18	D-imyoi-inositol 145-trisphosphate biosynthesis	0.02	https://metacyc.org/META/NEW-IMAGE?type=PATHWAY&object=PWY-6351

On the other hand, we also screened for the pathways that were most informative as biomarkers (AUC>0.75) for the maximum number of cancer types. Top 25 most informative biomarker pathways are shown on Table 2.

**Table 2:** Top 25 molecular pathways sorted by the number of cancer types where *PI* score serves as a good biomarker distinguishing from the other fourteen localizations (AUC>0.75).

**#**	**Pathway name (according to the source pathway database)**	**Cancers**	**Reference**
1	KEGG Pathways in cancer Main Pathway	6	https://www.genome.jp/kegg-bin/show_pathway?hsa05200
2	*AKT* Signaling Pathway	5	https://www.qiagen.com/us/shop/genes-and-pathways/pathway-details/?pwid=23
3	*cAMP* Pathway	5	https://www.qiagen.com/br/shop/genes-and-pathways/pathway-details/?pwid=76
4	*ILK* Signaling Pathway	5	https://www.qiagen.com/dk/shop/genes-and-pathways/pathway-details/?pwid=246
5	*ILK* Signaling Pathway Cytoskeletal Adhesion Complexes	5	https://www.qiagen.com/dk/shop/genes-and-pathways/pathway-details/?pwid=246
6	KEGG Neuroactive ligand receptor interaction Main Pathway	5	https://www.genome.jp/kegg-bin/show_pathway?map=hsa04080&show_description=show
7	*PTEN* Pathway Adhesion or Migration	5	https://www.qiagen.com/us/shop/genes-and-pathways/pathway-details/?pwid=375
8	*PTEN* Pathway Angiogenesis and Tumorigenesis	5	https://www.qiagen.com/us/shop/genes-and-pathways/pathway-details/?pwid=375
9	*PTEN* Pathway Ca2+ Signaling	5	https://www.qiagen.com/us/shop/genes-and-pathways/pathway-details/?pwid=375
10	*ERK* Signaling Pathway	4	https://www.qiagen.com/fr/shop/genes-and-pathways/pathway-details/?pwid=162
11	*ILK* Signaling Pathway Epithelial Mesenchymal Transition Tubulo-Interstitial Fibrosis	4	https://www.qiagen.com/dk/shop/genes-and-pathways/pathway-details/?pwid=246
12	*ILK* Signaling Pathway Migration Vasculogenesis	4	https://www.qiagen.com/dk/shop/genes-and-pathways/pathway-details/?pwid=246
13	KEGG *ECM* receptor interaction Main Pathway	4	https://www.genome.jp/kegg-bin/show_pathway?hsa04512
14	KEGG HTLV I infection Main Pathway	4	https://www.genome.jp/kegg-bin/show_pathway?hsa05166
15	KEGG *MAPK* signaling Main Pathway	4	https://www.genome.jp/kegg/pathway/hsa/hsa04010.html
16	KEGG Olfactory transduction Main Pathway	4	https://www.genome.jp/kegg-bin/show_pathway?map=hsa04740&show_description=show
17	KEGG Protein digestion and absorption Main Pathway	4	https://www.genome.jp/kegg-bin/show_pathway?map=hsa04974&show_description=show
18	KEGG Sphingolipid signaling Main Pathway	4	https://www.genome.jp/kegg-bin/show_pathway?map=hsa04071&show_description=show
19	*MAPK* Signaling Pathway	4	https://www.qiagen.com/us/shop/genes-and-pathways/pathway-details/?pwid=282
20	*MTOR* Pathway	4	https://www.qiagen.com/us/shop/genes-and-pathways/pathway-details/?pwid=304
21	NCI Beta1 integrin cell surface interactions Main Pathway	4	http://www.pathwaycommons.org/pc/record2.do?id=517095
22	*p38* Signaling Pathway	4	https://www.qiagen.com/mx/shop/genes-and-pathways/pathway-details/?pwid=337
23	*PAK* Pathway	4	https://www.qiagen.com/us/shop/genes-and-pathways/pathway-details/?pwid=342
24	*PTEN* Pathway	4	https://www.qiagen.com/us/shop/genes-and-pathways/pathway-details/?pwid=375
25	Ras Pathway	4	https://www.qiagen.com/no/shop/genes-and-pathways/pathway-details/?pwid=383

### Pairwise Comparison Biomarkers

**Figure 4:**
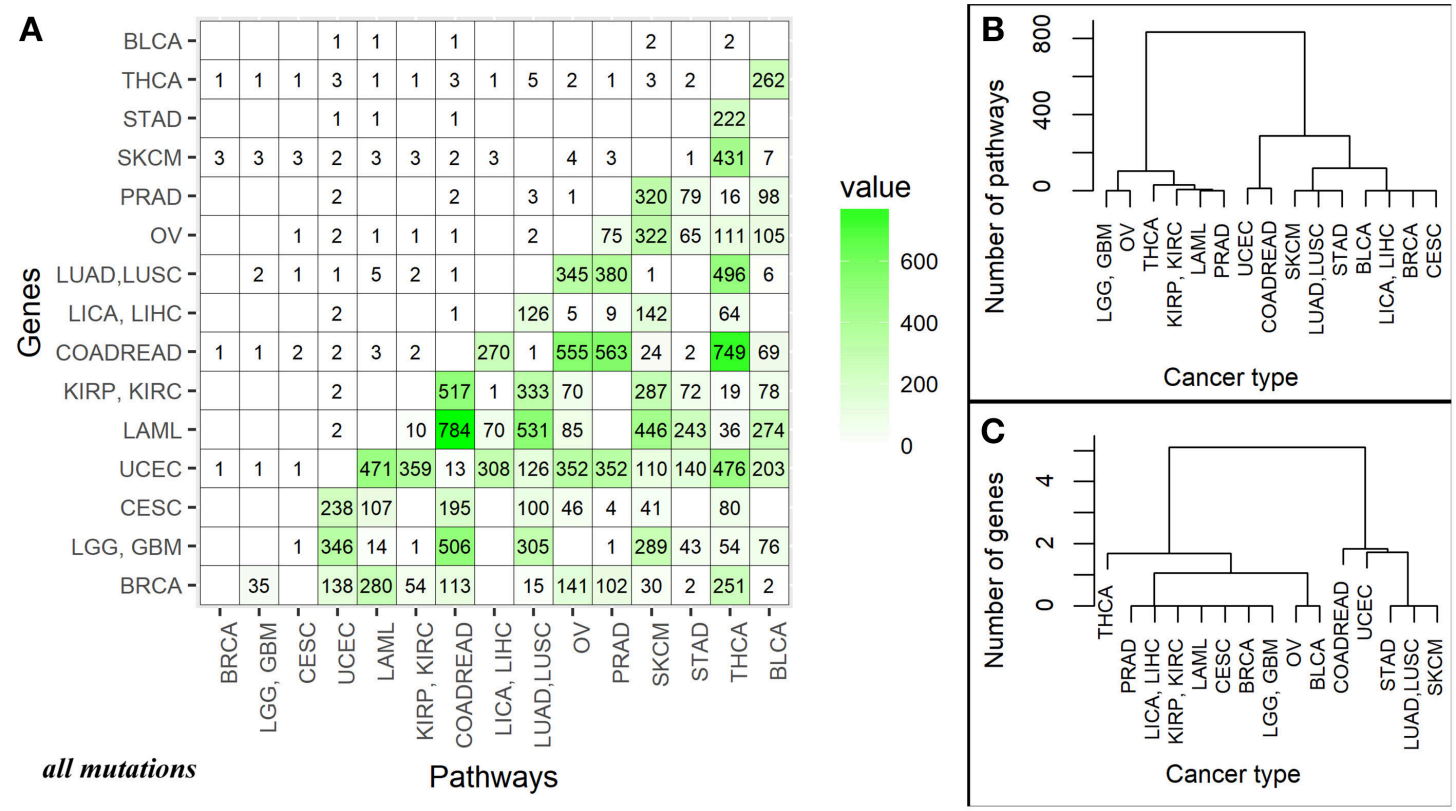
**(A)** Data matrix of high quality (AUC>0.75) biomarkers for pairwise comparisons between the different cancer localizations calculated based on all mutations. The cancer types are abbreviated as follows: BRCA, breast invasive carcinoma; LGG, brain lower grade glioma; GBM, glioblastoma multiforme; CESC, cervical squamous cell carcinoma and endocervical adenocarcinoma; UCEC, uterine corpus endometrial carcinoma; LAML, acute myeloid leukemia; KIRP, kidney renal papillary cell carcinoma; KIRC, kidney renal clear cell carcinoma; COADREAD, colorectal cancer; LICA, liver cancer; LIHC, liver hepatocellular carcinoma; LUAD, lung adenocarcinoma; LUSC, lung squamous cell carcinoma; OV, ovarian serous cystadenocarcinoma; PRAD, prostate adenocarcinoma; SKCM, skin cutaneous melanoma; STAD, stomach adenocarcinoma; THCA, thyroid carcinoma; BLCA, bladder urothelial carcinoma. The lower triangle shows numbers of high-quality biomarkers for pathway-based data (*PI*); the upper triangle—for individual gene-based mutation data (*nMR*). The intersection of cancer localization terms indicates number of the effective biomarkers for the respective comparison. **(B)** Cluster dendrogram built for the fifteen cancer types based on mutation biomarker (*PI)* data for all mutations. Number of biomarkers was used as the distance metric. **(C)** Cluster dendrogram built for the fifteen cancer types based on mutation biomarker (*nMR)* data for all mutations. Number of biomarkers was used as the distance metric.

The number of high quality biomarkers identified in pairwise comparisons can be characteristic of tumor mutational landscapes and their relative similarities. For example, when two cancer types under comparison have little or no specific biomarkers, this suggests small differences in their mutation profiles. In contrast, high number of biomarkers would mean more distinct mutation profiles. Based on the numbers of high quality *ROC AUC* biomarkers, a distance matrix can be created for all the tumor localizations under comparison, and a clustering dendrogram can be built. In this study, we used pairwise comparisons to analyze common features and clustering of 15 tumor types. The distance matrix was built separately for the gene (*nMR*) and the pathway (*PI*) mutation biomarkers (Figure 4A and Supplementary Image 2A for all and truncating mutations, respectively).

For all mutations, in a series of pairwise comparisons we found that the number of biomarkers that distinguish between the two cancer types varies greatly depending on the localizations compared (Figure 4A). The number of *PI* roughly two orders of magnitude per instance exceeded the number of *nMR* biomarkers (Figure 4A).

There was also an overall correlation between these numbers (correlation 0.69, *p*-value = 3.677e-16), for example, all fourteen comparisons having no good biomarkers according to *PI* values, also had no *nMR* biomarkers there (Figure 4A). The numbers of biomarkers differed for *PI* up to 784 and for *nMR*—only up to 5 per instance. Provided obviously higher number of the effective biomarkers, the pathway-based approach can be regarded beneficial and much more informative than the gene-based mutation analysis.

For the truncating mutations, we found that the number of respective pathway biomarkers was also significantly higher (up to 43 pathway biomarkers vs. only one gene biomarker), but they were identified in a smaller group of cancer types (Supplementary Image 2A). Nevertheless, the correlation between gene and pathway pairwise comparisons was 0.634, *p*-value = 3.62e-13.

### Clustering Dendrograms of Cancer Localizations

Taking number of molecular pathways with significant AUC as a metric of the proximity between cancer types, we built clustering dendrograms for the 15 investigated cancer types. The dendrograms generated for the different cancer types differed considerably for the *nMR* and *PI* data. We focused on the results based on all mutations, because the dendrograms built using only truncating mutations were not biologically informative (Supplementary Images 2B,C).

First of all, the *nMR*-based tree had lower number of major clades (three vs. four for the *PI* data tree). Second, distances between the cancer localizations were more degenerated on the *nMR* tree (Figures 4B,C). These features may reflect the approximately two orders of magnitude lower numbers of biomarkers used to construct dendrograms identified for the *nMR* data. Clade compositions were largely similar between both types of tree, but the above considerations suggest in favor of using pathway- rather than gene-specific tree based on mutation data.

Interestingly, positions on the clades of all the dendrograms were not linked with the anatomical proximities of the respective localizations in human body. This suggests that accumulations of characteristic mutations in cancers are following complex mechanisms that are not yet completely understood.

## Discussion

Bioinformatic approaches based on measuring of molecular pathway activation were efficient in finding biomarkers using high throughput proteomics (25), mRNA (27, 51), microRNA (26) and even transcription factor binding site data (52). Here, we applied molecular pathway scoring approach to large scale mutation data. For the first time, we developed reliable universal method of measuring mutation enrichment of molecular pathways. It should be noted that the idea of collapsing mutation data has been already reflected in several previous studies. For example, bioinformatic tool BioBin overcomes sparsity of data by combining mutations into bins at the levels of molecular pathways, protein families, evolutionary conversed regions and regulatory regions (53, 54). An alternative approach for the same has been provided by the method Network regularized sparse non-negative TRI matrix factorization for PATHway identification using known molecular pathways and gene interaction networks (55). Unlike previous methods, our approach focuses on generation of universal parameters that objectively assess the mutation load of a molecular pathway. Previous approaches evaluated mutation load only based on presence or absence of a mutation, not considering number of gene products-pathway participants and lengths of their DNA coding sequences, which hindered accurate comparison of pathways. These major problems were addressed by the current *PI* approach, which provides clear, simple and reliable universal measure of mutation burden of molecular pathways.

We adopted this method for finding mutation biomarkers of cancers. On the example of 5,956 exome sequencing profiles of different cancer patients we showed at least two order of magnitude superior performance of the pathway instability scoring compared to the single gene-based approach.

In the current approach, we did not classify the effects of the different mutations on the pathway activities because only a minor fraction of the mutations identified has been experimentally characterized in terms of its impact on the protein and pathway functionalities. However, further accumulation of these data on a high throughput basis will make it possible to improve the PI calculation by adding the specific coefficients reflecting effect of every individual mutation on the respective protein. To assess stability of *PI* scoring, we also tested a version of this method considering only a minor fraction of truncating mutations that most likely led to the loss of gene function. Truncating mutations have demonstrated the same trends as the total fraction of mutations, thus confirming *PI* scoring robustness.

This method can be easily translated to comparisons of every sets of human exome or complete genome data. To this end, for every sample, mutations should be identified for the genes participating in the molecular pathways under investigation (1,748 pathways including 8,543 genes in this study). Pathway instability (PI) scores are then calculated showing relative mutation burden of each pathway in every biosample. These findings can be valuable *per se* for better understanding of the individual mechanisms of carcinogenesis. Furthermore, ROC AUC test can be next applied to the PI data to identify reliable biomarkers of the sample groups under comparison. All these procedures can be done by using publicly available bioinformatic tools, and the gene compositions of 1,748 molecular pathways required for PI calculation are available in the respective databases (35–40).

Taken together, our data suggest that in addition to better understanding of fundamental tissue-specific mechanisms of carcinogenesis, molecular pathway approach can be beneficial in finding reliable tumor type-specific biomarkers for identification of tumor origin in the low- or non-differentiated tumor histotypes. *Pathway instability* (*PI)* mutation data can be used as the additional criteria for differential diagnostics in oncology. Tumor relationship based on the pathway specific genetic signatures such as those shown on Figure 4C may help optimize design of the basket clinical trials. This may be also beneficial to help to predict common patterns in response to drugs and different treatment regimens.

Although a major focus of this study was made on the specific deviations in PI between the cancer types, we could also identify molecular pathways that were strongly mutated in all the cancer types, as previously predicted in the literature (19, 21).

Finally, we provide the list of 660 marker molecular pathways that distinguish between the major human cancer types. This list can be helpful for better understanding molecular grounds of carcinogenesis and for further investigations in molecular oncology and drug development.

## Data Availability Statement

The datasets analyzed in this study are available in COSMIC public repository (COSMICv76; CosmicGenomeScreensMutantExport.tsv.gz, https://cancer.sanger.ac.uk/cosmic/download).

## Author Contributions

MZ developed algorithms, did mutation/Pathway instability analyses, planned the research, and wrote the manuscript. SR planned the research, extracted, and filtered cancer mutation data. MS completed the molecular pathway database. NB developed algorithms, did statistical analyses, and planned the research. AB developed algorithms, planned the research, and wrote the manuscript.

### Conflict of Interest Statement

The authors declare that the research was conducted in the absence of any commercial or financial relationships that could be construed as a potential conflict of interest.

## Supplementary Material

Supplementary Data Sheet 1 *nMR* scores for 19 872 genes in 5956 tumor samples. The scores were calculated using all mutations data.

Supplementary Data Sheet 2 *nMR* scores for 16 760 genes in 5297 tumor samples. The scores were calculated using truncating mutations data.

Supplementary Data Sheet 3 *PI* scores for 1748 molecular pathways in 5956 tumor samples. The scores were calculated using all mutations data.

Supplementary Data Sheet 4 *PI* scores for 1748 molecular pathways in 5297 tumor samples. The scores were calculated using truncating mutations data.

Supplementary Table 1 Tumor localizations and numbers of tumor samples investigated for each cancer type, for all mutations.

Supplementary Table 2 Tumor localizations and numbers of tumor samples investigated for each cancer type, for only truncating mutation.

Supplementary Table 3 The database accession numbers and primary localizations of 5956 tumor samples.

Supplementary Table 4 The database accession numbers and primary localizations of 5297 tumor samples containing truncating mutations.

Supplementary Table 5 AUC values for comparisons of every separately taken tumor type (localization) versus all other tumor types, calculated for all mutations. The sheet “Pathway biomarkers” contains AUC values and AUC 95% confidence intervals for pathways having AUC > 0.75 in at least one of the comparisons. The sheet “Gene biomarkers” includes AUC values and AUC 95% confidence intervals for genes having AUC > 0.75 in at least one of the comparisons.

Supplementary Table 6 AUC values for comparisons of every separately taken tumor type (localization) versus all other tumor types, calculated for truncating mutations. The sheet “Pathway biomarkers” contains AUC values and AUC 95% confidence intervals for pathways having AUC > 0.75 in at least one of the comparisons. The sheet “Gene biomarkers” includes AUC values and AUC 95% confidence intervals for genes having AUC > 0.75 in at least one of the comparisons.

Supplementary Table 7 List of tumor type-specific good-quality pathway biomarkers for eight tumor types, obtained using all mutations data. Every sheet corresponds to the primary localization of tumors and contains pathway names, AUC values and their 95% confidence interval (CI), mark about PI values of this tumor type relative to others, average PI scores for all cancer types, average PI for this cancer type. TCGA abbreviations of tumor types were used: uterine corpus endometrial carcinoma - UCEC, acute myeloid leukemia - LAML, kidney renal papillary cell carcinoma - KIRP, kidney renal clear cell carcinoma - KIRC, colorectal cancer - COADREAD, lung adenocarcinoma - LUAD, lung squamous cell carcinoma - LUSC, prostate adenocarcinoma - PRAD, skin cutaneous melanoma - SKCM, thyroid carcinoma - THCA. The data about all mutations was used.

Supplementary Table 8 List of colorectal cancer specific good-quality pathway biomarkers, obtained using truncating mutations data. The table contains pathway names, AUC values and their 95% confidence intervals (CI), note PI scores for colorectal cancer relative to other cancer types.

Supplementary Table 9 1145 pathways with AUC < 0.7 for all tumor types in comparisons of every separately taken tumor type (localization) versus all other tumor types. All mutations data were used.

Supplementary Image 1 Correlations with gene coding DNA sequence lengths. **(A)** Correlation of *Mutation rates (MR)* and gene CDS lengths calculated for 5956 samples from fifteen tumor localizations, for all mutations. **(B)** Correlation of *Normalized mutation rates (nMR)* and gene CDS lengths calculated for the same biosamples, for all mutations. **(C)** Correlation of *Mutation rates (MR)* and gene CDS lengths calculated for 5297 samples from fifteen tumor localizations, for truncating mutations. **(D)** The correlation of *Normalized mutation rates (nMR)* and gene CDS lengths for the same biosamples, for truncating mutations.

Supplementary Image 2 **(A)** Data matrix of high quality (AUC > 0.75) biomarkers for pairwise comparisons between different cancer types based on truncating mutations only. The cancer types are abbreviated as follows: breast invasive carcinoma - BRCA, brain lower grade glioma - LGG, glioblastoma multiforme - GBM, cervical squamous cell carcinoma and endocervical adenocarcinoma - CESC, uterine corpus endometrial carcinoma - UCEC, acute myeloid leukemia - LAML, kidney renal papillary cell carcinoma - KIRP, kidney renal clear cell carcinoma - KIRC, colorectal cancer - COADREAD, liver cancer - LICA, liver hepatocellular carcinoma - LIHC, lung adenocarcinoma - LUAD, lung squamous cell carcinoma - LUSC, ovarian serous cystadenocarcinoma - OV, prostate adenocarcinoma - PRAD, skin cutaneous melanoma - SKCM, stomach adenocarcinoma - STAD, thyroid carcinoma - THCA, bladder urothelial carcinoma - BLCA. The lower triangle shows numbers of good biomarkers for pathway-based data *(PI)*; the upper triangle - for individual gene-based mutation data *(nMR)*. **(B)** Clustering dendrogram built for the fifteen cancer types based on mutation biomarker *(PI)* data for truncating mutations. Number of biomarkers was used as the distance metric. **(C)** Clustering dendrogram built for the above fifteen cancer types using mutation biomarker *(nMR)* data for truncating mutations. Number of biomarkers was used as the distance metric.

## Abbreviations

CDS length: coding DNA sequence length
COSMIC: Catalog Of Somatic Mutations In Cancer
ICGC: International Cancer Genome Consortium
MR: Mutation rate
nMR: Normalized mutation rate
PAS: pathway activation strength
PCA: principal component analysis
PI: Pathway instability
ROC AUC: Receiver Operator Characteristics Area Under the Curve
TCGA: The Cancer Genome Atlas.

## References

[1] Sieber O Heinimann K Tomlinson I. Genomic stability and tumorigenesis. Semin Cancer Biol. (2005) 15:61–6. 10.1016/j.semcancer.2004.09.005 15613289

[2] Jakóbisiak M Lasek W Gołab J. Natural mechanisms protecting against cancer. Immunol Lett. (2003) 90:103–22. 10.1016/j.imlet.2003.08.005 14687712

[3] Cha HJ Yim H. The accumulation of DNA repair defects is the molecular origin of carcinogenesis. Tumor Biol. (2013) 34:3293–302. 10.1007/s13277-013-1038-y 23907577

[4] Vogelstein B Papadopoulos N Velculescu VE Zhou S Diaz LA Kinzler KW. Cancer genome landscapes. Science (2013) 339:1546–58. 10.1126/science.1235122 23539594 PMC3749880

[5] Kandoth C McLellan MD Vandin F Ye K Niu B Lu C. Mutational landscape and significance across 12 major cancer types. Nature (2013) 502:333–9. 10.1038/nature12634 24132290 PMC3927368

[6] Bignell GR Greenman CD Davies H Butler AP Edkins S Andrews JM. Signatures of mutation and selection in the cancer genome. Nature (2010) 463:893–8. 10.1038/nature08768 20164919 PMC3145113

[7] Campbell PJ Yachida S Mudie LJ Stephens PJ Pleasance ED Stebbings LA. The patterns and dynamics of genomic instability in metastatic pancreatic cancer. Nature (2010) 467:1109–13. 10.1038/nature09460 20981101 PMC3137369

[8] International Cancer Genome Consortium TICG Hudson TJ Anderson W Artez A Barker AD Bell C International network of cancer genome projects. Nature (2010) 464:993–8. 10.1038/nature08987 20393554 PMC2902243

[9] Rafiq S Khan S Tapper W Collins A Upstill-Goddard R Gerty S. A genome wide meta-analysis study for identification of common variation associated with breast cancer prognosis. PLoS ONE (2014) 9:e101488. 10.1371/journal.pone.0101488 25526632 PMC4272267

[10] Mitra AP Lerner SP. Potential role for targeted therapy in muscle-invasive bladder cancer: lessons from the cancer genome atlas and beyond. Urol Clin North Am. (2015) 42:201–15. 10.1016/j.ucl.2015.01.003 25882562

[11] Keshaviah A Dellapasqua S Rotmensz N Lindtner J Crivellari D Collins J. CA15-3 and alkaline phosphatase as predictors for breast cancer recurrence: a combined analysis of seven International Breast Cancer Study Group trials. Ann Oncol. (2006) 18:701–8. 10.1093/annonc/mdl492 17237474

[12] Krishnan STM Philipose Z Rayman G. Lesson of the week: hypothyroidism mimicking intra-abdominal malignancy. BMJ (2002) 325:946–7. 10.1136/bmj.325.7370.946 12399347 PMC1124444

[13] Sonnenschein C Soto AM. Theories of carcinogenesis: an emerging perspective. Semin Cancer Biol. (2008) 18:372–7. 10.1016/j.semcancer.2008.03.012 18472276 PMC2730644

[14] Røsland GV Engelsen AST. Novel points of attack for targeted cancer therapy. Basic Clin Pharmacol Toxicol. (2015) 116:9–18. 10.1111/bcpt.12313 25154903 PMC4309509

[15] Duffy MJ Clinical use of tumor biomarkers: an overview. Klin Biochem Metab. (2017) 25:157–61. Available online at: http://www.cskb.cz/res/file/KBM-pdf/2017/2017-4/KBM-2017-4-Duffy-157.pdf (Accessed August 29, 2018).

[16] Sowter HM Ashworth A. BRCA1 and BRCA2 as ovarian cancer susceptibility genes. Carcinogenesis (2005) 26:1651–6. 10.1093/carcin/bgi136 15917310

[17] Thériault C Pinard M Comamala M Migneault M Beaudin J Matte I. MUC16 (CA125) regulates epithelial ovarian cancer cell growth, tumorigenesis and metastasis. Gynecol Oncol. (2011) 121:434–43. 10.1016/j.ygyno.2011.02.020 21421261

[18] Zhang Q Burdette JE Wang JP. Integrative network analysis of TCGA data for ovarian cancer. BMC Syst Biol. (2014) 8:1338. 10.1186/s12918-014-0136-9 25551281 PMC4331442

[19] Chong ML Loh M Thakkar B Pang B Iacopetta B Soong R. Phosphatidylinositol-3-kinase pathway aberrations in gastric and colorectal cancer: meta-analysis, co-occurrence and ethnic variation. Int J Cancer (2014) 134:1232–8. 10.1002/ijc.28444 23960014

[20] Li H Zeng J Shen K. PI3K/AKT/mTOR signaling pathway as a therapeutic target for ovarian cancer. Arch Gynecol Obstet. (2014) 290:1067–78. 10.1007/s00404-014-3377-3 25086744

[21] Toren P Zoubeidi A. Targeting the PI3K/Akt pathway in prostate cancer: challenges and opportunities. Int J Oncol. (2014) 45:1793–801. 10.3892/ijo.2014.2601 25120209

[22] Buzdin AA Zhavoronkov AA Korzinkin MB Venkova LS Zenin AA Smirnov PY. Oncofinder, a new method for the analysis of intracellular signaling pathway activation using transcriptomic data. Front Genet. (2014) 5:55. 10.3389/fgene.2014.00055 24723936 PMC3971199

[23] Ozerov IV Lezhnina KV Izumchenko E Artemov AV Medintsev S Vanhaelen Q Aliper A. *In silico* pathway activation network decomposition analysis (iPANDA) as a method for biomarker development. Nat Commun. (2016) 7:13427. 10.1038/ncomms13427 27848968 PMC5116087

[24] Buzdin A Sorokin M Garazha A Sekacheva M Kim E Zhukov N. Molecular pathway activation - new type of biomarkers for tumor morphology and personalized selection of target drugs. Semin Cancer Biol. (2018) 53:110–24. 10.1016/j.semcancer.2018.06.003 29935311

[25] Borisov N Suntsova M Sorokin M Garazha A Kovalchuk O Aliper A. Data aggregation at the level of molecular pathways improves stability of experimental transcriptomic and proteomic data. Cell Cycle (2017) 16:1810–23. 10.1080/15384101.2017.1361068 28825872 PMC5628641

[26] Artcibasova AV Korzinkin MB Sorokin MI Shegay PV Zhavoronkov AA Gaifullin N. MiRImpact, a new bioinformatic method using complete microRNA expression profiles to assess their overall influence on the activity of intracellular molecular pathways. Cell Cycle (2016) 15:689–98. 10.1080/15384101.2016.1147633 27027999 PMC4845938

[27] Aliper AM Korzinkin MB Kuzmina NB Zenin AA Venkova LS Smirnov PY. Mathematical justification of expression-based Pathway Activation Scoring (PAS). Methods Mol Biol. (2017) 1613:31–51. 10.1007/978-1-4939-7027-8_3 28849557

[28] Buzdin AA Prassolov V Zhavoronkov AA Borisov NM. Bioinformatics meets biomedicine: oncofinder, a quantitative approach for interrogating molecular pathways using gene expression data. Methods Mol Biol. (2017) 1613:53–83. 10.1007/978-1-4939-7027-8_4 28849558

[29] Borisov NM Terekhanova NV Aliper AM Venkova LS Smirnov PY Roumiantsev S. Signaling pathways activation profiles make better markers of cancer than expression of individual genes. Oncotarget (2014) 5:10198–205. 10.18632/oncotarget.2548 25415353 PMC4259415

[30] Buzdin AA Zhavoronkov AA Korzinkin MB Roumiantsev SA Aliper AM Venkova LS. The OncoFinder algorithm for minimizing the errors introduced by the high-throughput methods of transcriptome analysis. Front Mol Biosci. (2014) 1:8. 10.3389/fmolb.2014.00008 25988149 PMC4428387

[31] Forbes SA Beare D Gunasekaran P Leung K Bindal N Boutselakis H. COSMIC: exploring the world's knowledge of somatic mutations in human cancer. Nucleic Acids Res. (2015) 43:D805–11. 10.1093/nar/gku1075 25355519 PMC4383913

[32] Forbes SA Beare D Boutselakis H Bamford S Bindal N Tate J. COSMIC: somatic cancer genetics at high-resolution. Nucleic Acids Res. (2017) 45:D777–83. 10.1093/nar/gkw1121 27899578 PMC5210583

[33] Home - *The Cancer Genome Atlas - Cancer Genome - TCGA* Available online at: https://cancergenome.nih.gov/ (Accessed September 19, 2018).

[34] Tomczak K Czerwinska P Wiznerowicz M. The Cancer Genome Atlas (TCGA): an immeasurable source of knowledge. Contemp Oncol. (2015) 19:A68–77. 10.5114/wo.2014.47136 25691825 PMC4322527

[35] Croft D Mundo AF Haw R Milacic M Weiser J Wu G. The Reactome pathway knowledgebase. Nucleic Acids Res. (2014) 42:D472–7. 10.1093/nar/gkt1102 24243840 PMC3965010

[36] Schaefer CF Anthony K Krupa S Buchoff J Day M Hannay T. PID: the pathway interaction database. Nucleic Acids Res. (2009) 37:D674–9. 10.1093/nar/gkn653 18832364 PMC2686461

[37] Kanehisa M Goto S. KEGG: kyoto encyclopedia of genes and genomes. Nucleic Acids Res. (2000) 28:27–30. 10.1093/nar/28.1.27 10592173 PMC102409

[38] Romero P Wagg J Green ML Kaiser D Krummenacker M Karp PD. Computational prediction of human metabolic pathways from the complete human genome. Genome Biol. (2004) 6:R2. 10.1186/gb-2004-6-1-r2 15642094 PMC549063

[39] Nishimura D BioCarta. Biotech Softw Internet Rep. (2001) 2:117–120. 10.1089/152791601750294344

[40] QIAGEN - *Sample to Insight* Available online at: https://www.qiagen.com/us/shop/genes-and-pathways/pathway-central/ (Accessed September 19, 2018).

[41] Murtagh F Legendre P Ward's Hierarchical Agglomerative Clustering Method: which algorithms implement ward's criterion? J Classif. (2014) 31:274–95. 10.1007/s00357-014-9161-z

[42] Green DM Swets JA Signal Detection Theory and Psychophysics. New York, NY: Wiley (1966).

[43] Boyd JC. Mathematical tools for demonstrating the clinical usefulness of biochemical markers. Scand J Clin Lab Invest Suppl. (1997) 227:46–63. 10.1080/00365519709168308 9127468

[44] Chen L Zhou Y Tang X Yang C Tian Y Xie R EGFR mutation decreases FDG uptake in non-small cell lung cancer via the NOX4/ROS/GLUT1 axis. Int J Oncol. (2018) 54:370–80. 10.3892/ijo.2018.4626 30431083

[45] Tanioka M Fan C Parker JS Hoadley KA Hu Z Li Y. Integrated analysis of RNA and DNA from the phase III trial CALGB 40601 identifies predictors of response to trastuzumab-based neoadjuvant chemotherapy in HER2-positive breast cancer. Clin Cancer Res. (2018) 24:5292–304. 10.1158/1078-0432.CCR-17-3431 30037817 PMC6214737

[46] Liu T Cheng G Kang X Xi Y Zhu Y Wang K. Noninvasively evaluating the grading and IDH1 mutation status of diffuse gliomas by three-dimensional pseudo-continuous arterial spin labeling and diffusion-weighted imaging. Neuroradiology (2018) 60:693–702. 10.1007/s00234-018-2021-5 29777252

[47] Fodde R. The APC gene in colorectal cancer. Eur J Cancer (2002) 38:867–71. 10.1016/S0959-8049(02)00040-0 11978510

[48] Risinger JI Hayes K Maxwell GL Carney ME Dodge RK Barrett JC. PTEN mutation in endometrial cancers is associated with favorable clinical and pathologic characteristics. Clin Cancer Res. (1998) 4:3005–10. 9865913

[49] Cohen Y Xing M Mambo E Guo Z Wu G Trink B. BRAF mutation in papillary thyroid carcinoma. JNCI J Natl Cancer Inst. (2003) 95:625–7. 10.1093/jnci/95.8.625 12697856

[50] Lawrence MS Stojanov P Polak P Kryukov GV Cibulskis K Sivachenko A. Mutational heterogeneity in cancer and the search for new cancer-associated genes. Nature (2013) 499:214–8. 10.1038/nature12213 23770567 PMC3919509

[51] Zhu Q Izumchenko E Aliper AM Makarev E Paz K Buzdin AA. Pathway activation strength is a novel independent prognostic biomarker for cetuximab sensitivity in colorectal cancer patients. Hum genome Var. (2015) 2:15009. 10.1038/hgv.2015.9 27081524 PMC4785572

[52] Nikitin D Penzar D Garazha A Sorokin M Tkachev V Borisov N. Profiling of human molecular pathways affected by retrotransposons at the level of regulation by transcription factor proteins. Front Immunol. (2018) 9:30. 10.3389/fimmu.2018.00030 29441061 PMC5797644

[53] Moore CB Wallace JR Frase AT Pendergrass SA Ritchie MD. BioBin: a bioinformatics tool for automating the binning of rare variants using publicly available biological knowledge. BMC Med Genomics (2013) 6(Suppl. 2):S6. 10.1186/1755-8794-6-S2-S6 23819467 PMC3654874

[54] Kim D Li R Dudek SM Wallace JR Ritchie MD Binning somatic mutations based on biological knowledge for predicting survival: an application in renal cell carcinoma. Pac Symp Biocomput. (2015)20:96–107. 10.1142/9789814644730_0011 PMC4299944 25592572

[55] Park S Kim SJ Yu D Peña-Llopis S Gao J Park JS. An integrative somatic mutation analysis to identify pathways linked with survival outcomes across 19 cancer types. Bioinformatics (2016) 32:1643–51. 10.1093/bioinformatics/btv692 26635139 PMC4892411

