# Supplementary figures and images for: Pathway Instability Is an Effective New Mutation-Based Type of Cancer Biomarkers
Source: Front Oncol. 2019 Jan 4;8:658. doi: 10.3389/fonc.2018.00658 (PMC6328788; doi:10.3389/fonc.2018.00658)

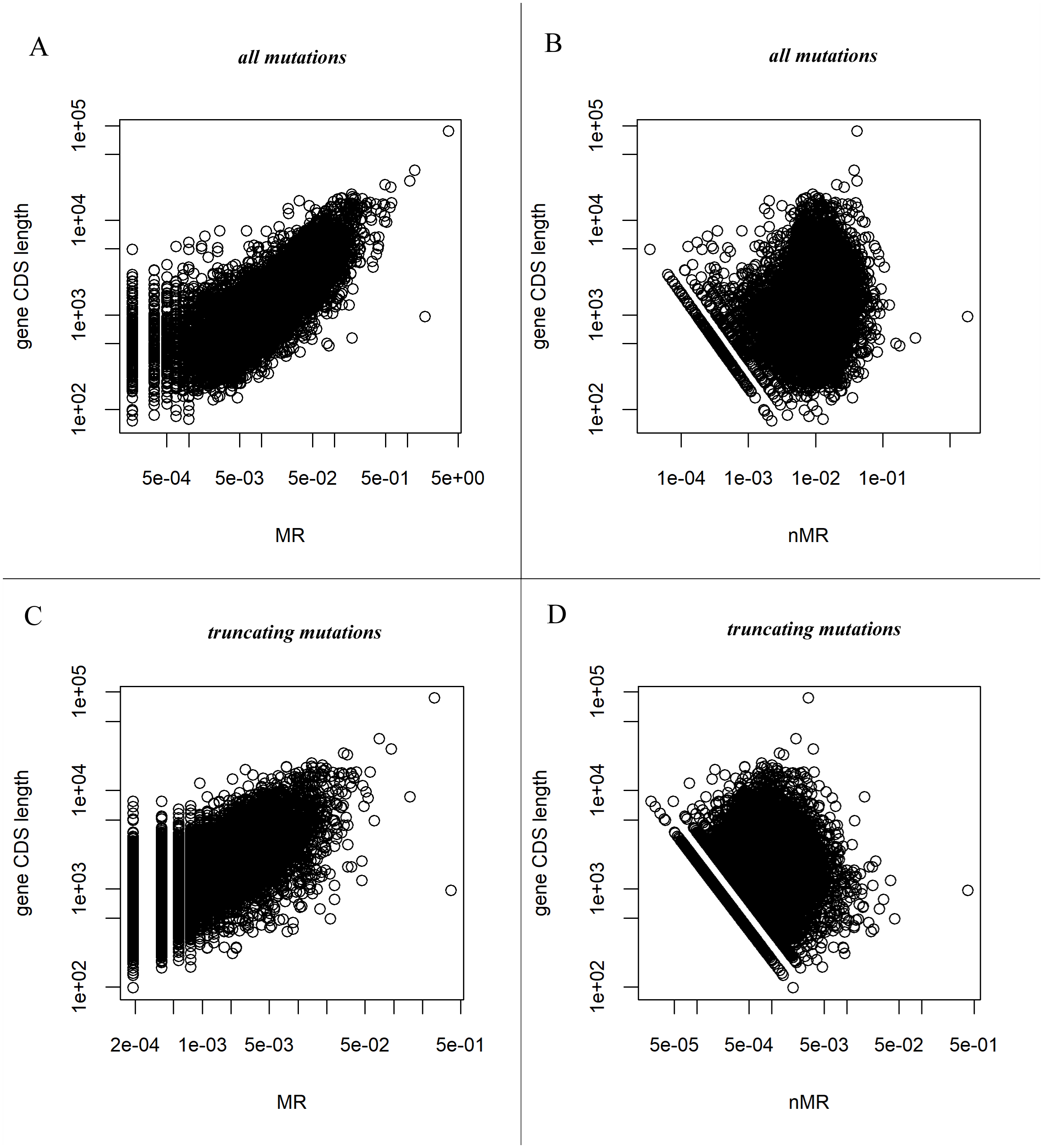

Supplement: Supplementary Image 1 — Correlations with gene coding DNA sequence lengths. (A) Correlation of Mutation rates (MR) and gene CDS lengths calculated for 5956 samples from fifteen tumor localizations, for all mutations. (B) Correlation of Normalized mutation rates (nMR) and gene CDS lengths calculated for the same biosamples, for all mutations. (C) Correlation of Mutation rates (MR) and gene CDS lengths calculated for 5297 samples from fifteen tumor localizations, for truncating mutations. (D) The correlation of Normalized mutation rates (nMR) and gene CDS lengths for the same biosamples, for truncating mutations. [file Image_1.TIF]

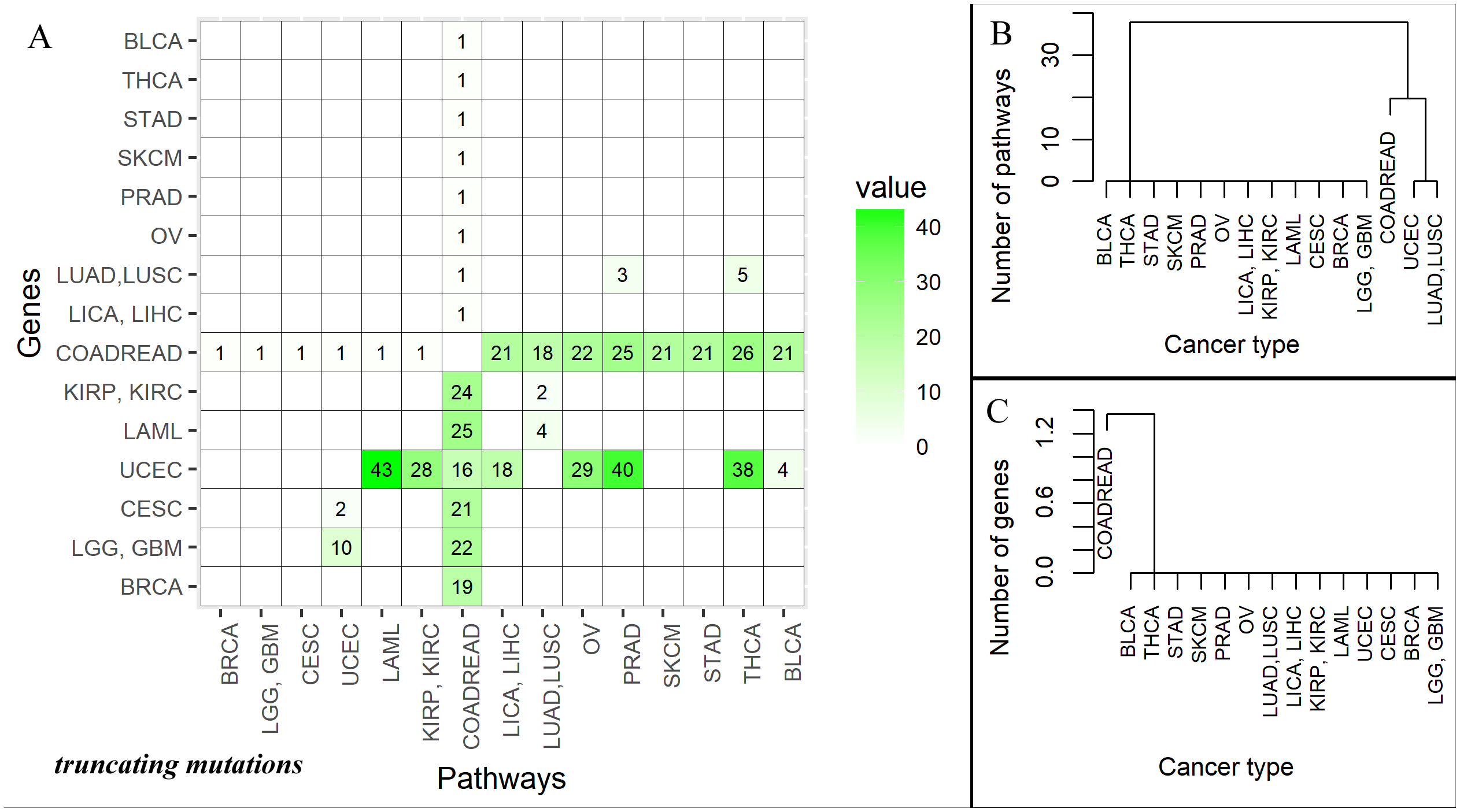

Supplement: Supplementary Image 2 — (A) Data matrix of high quality (AUC > 0.75) biomarkers for pairwise comparisons between different cancer types based on truncating mutations only. The cancer types are abbreviated as follows: breast invasive carcinoma - BRCA, brain lower grade glioma - LGG, glioblastoma multiforme - GBM, cervical squamous cell carcinoma and endocervical adenocarcinoma - CESC, uterine corpus endometrial carcinoma - UCEC, acute myeloid leukemia - LAML, kidney renal papillary cell carcinoma - KIRP, kidney renal clear cell carcinoma - KIRC, colorectal cancer - COADREAD, liver cancer - LICA, liver hepatocellular carcinoma - LIHC, lung adenocarcinoma - LUAD, lung squamous cell carcinoma - LUSC, ovarian serous cystadenocarcinoma - OV, prostate adenocarcinoma - PRAD, skin cutaneous melanoma - SKCM, stomach adenocarcinoma - STAD, thyroid carcinoma - THCA, bladder urothelial carcinoma - BLCA. The lower triangle shows numbers of good biomarkers for pathway-based data (PI); the upper triangle - for individual gene-based mutation data (nMR). (B) Clustering dendrogram built for the fifteen cancer types based on mutation biomarker (PI) data for truncating mutations. Number of biomarkers was used as the distance metric. (C) Clustering dendrogram built for the above fifteen cancer types using mutation biomarker (nMR) data for truncating mutations. Number of biomarkers was used as the distance metric. [file Image_2.TIF]
